# Tissue-Specific Angiogenic Responses to Exercise: Mechanisms and Research Advances

**DOI:** 10.3390/biom16091340

**Published:** 2026-09-15

**Authors:** Xingwen Zheng, Zhijian Rao, Zhitong Sun, Yibing Lu, Lifang Zheng, Yu Feng

**Affiliations:** 1 College of Physical Education, Shanghai University, Shanghai 200444, China; zxw2003@shu.edu.cn (X.Z.); 1287635496@shu.edu.cn (Z.S.); luyibing0513@shu.edu.cn (Y.L.); 2 College of Physical Education, Shanghai Normal University, Shanghai 200234, China; raoz19@shnu.edu.cn; 3 Exercise Biological Center, China Institute of Sport Science, Beijing 100061, China; 4 Department of Critical Care Medicine, Shanghai Children’s Hospital, School of Medicine, Shanghai Jiao Tong University, Shanghai 200062, China; 5 Institute of Pediatric Infection, Immunity and Intensive Care Medicine, Shanghai Jiao Tong University, Shanghai 200062, China

**Keywords:** exercise, angiogenesis, tissue-specific, VEGF, skeletal muscle, brain, heart

## Abstract

Exercise is a pivotal non-pharmacological intervention for enhancing overall health, with a key underlying mechanism being the induction of adaptive angiogenesis across multiple organ systems. This narrative review discusses recent advances in exercise-induced angiogenesis, with a particular focus on its tissue- and organ-specific manifestations and underlying molecular mechanisms. The available evidence indicates that exercise integrates diverse signals—including mechanical stress, metabolic shifts, and hypoxic or ischemic stress—to upregulate key angiogenic factors, such as vascular endothelial growth factor (VEGF), angiopoietin-1 (Ang-1) and angiopoietin-2 (Ang-2), and to activate conserved signaling pathways including hypoxia-inducible factor-1α (HIF-1α), phosphoinositide 3-kinase/protein kinase B (PI3K/Akt), and endothelial nitric oxide synthase (eNOS), thereby driving angiogenesis. This process exhibits tissue specificity: in skeletal muscle, it primarily enhances oxygen transport and metabolic efficiency; in bone, it couples with osteogenesis to maintain structural integrity; in the brain, it supports neuroplasticity and cognitive function; in the heart, it improves myocardial perfusion via angiogenesis and collateral remodeling (arteriogenesis); and in adipose tissue, it ameliorates the metabolic microenvironment. Furthermore, the mode and intensity of exercise, along with individual differences (such as age and health status), significantly influence the magnitude of its angiogenic effects. Accordingly, this review highlights the proposed role of exercise-induced angiogenesis in a range of conditions—including cardiovascular disease, sarcopenia, osteoporosis, neurodegenerative disorders, and metabolic syndrome—and we treat these relationships as potential mechanisms that require further validation rather than established therapeutic pathways. In the future, research should aim to define optimal exercise parameters, elucidate deeper molecular mechanisms, and investigate the translational potential of these insights for personalized clinical interventions.

## 1. Introduction

Angiogenesis is a biological process involving the formation of new capillaries from pre-existing vascular networks. As a crucial mechanism by which the body adapts to growth, development, and tissue repair, it helps to maintain internal homeostasis by constructing an effective microcirculation network that supplies tissues with oxygen and nutrients while removing metabolic waste [1,2]. Dysregulated angiogenesis represents a significant pathological factor in various chronic conditions, including cardiovascular disease and neurodegenerative disorders, making its targeted modulation a promising therapeutic strategy [3].

Regular physical exercise is a widely recognized, cost-effective, and accessible non-pharmacological intervention for disease prevention and health promotion. Its benefits extend beyond enhancing cardiopulmonary fitness and metabolic health to its profound effects on multiple physiological systems [4]. Mounting evidence indicates that exercise is a potent inducer of physiological angiogenesis. The increased metabolic demand and altered hemodynamics during exercise activate key signaling networks, such as the HIF-1α pathway, and markedly upregulate pivotal regulators like VEGF, thereby effectively driving new vessel formation and functional vascular adaptation [5,6].

Notably, the angiogenic response to exercise is not uniform but rather exhibits significant tissue and organ specificity. Due to differences in anatomical structure, functional requirements, and metabolic characteristics among different tissues, the vascular responses to identical exercise stimuli differ in their morphological manifestations, dominant pathways, and physiological significance [6]. This specificity is not limited to a single organ system: vascular beds across the body—including the liver [7], kidney [8], lung [9], skin [10], immune system [11], lymphatic tissues [12], and the tumor microenvironment [13]—can respond to exercise, albeit to varying extents and through distinct mechanisms. In this review, however, we focus on five representative tissues—skeletal muscle, bone, brain, heart, and adipose tissue—for which the evidence is best established and the physiological and clinical implications are most directly relevant. These five tissue systems were selected because they jointly span the principal contexts in which exercise exerts its health benefits: locomotor and metabolic function (skeletal muscle, adipose tissue), structural support and remodeling (bone), cognitive and neurological health (brain), and hemodynamic and ischemic adaptation (heart). Furthermore, dysregulated angiogenesis within these tissues is centrally implicated in the major chronic conditions that exercise is widely used to mitigate or manage, including sarcopenia [14], osteoporosis [15], Alzheimer’s disease [16], cardiovascular disease [17], and metabolic syndrome [18,19,20]. We therefore position this work as a focused review of selected, representative tissues rather than a comprehensive account of all exercise-responsive vascular beds; an exhaustive treatment of the remaining systems is beyond our present scope but represents an important direction for future synthesis.

For example, skeletal muscle primarily exhibits an increase in capillary density to optimize oxygen delivery; the brain exhibits functional coupling of neurovascular units to support cognitive activities; and the heart, bone, and adipose tissue each exhibit unique vascular remodeling patterns and regulatory mechanisms [21,22,23,24,25]. This specificity indicates that the pro-angiogenic effect of exercise results from the precise, coordinated, and differential activation of multiple signaling pathways within distinct tissue microenvironments.

Against this backdrop, in this narrative review, we aim to summarize recent advances in exercise-induced angiogenesis, focusing on its tissue-specific manifestations and underlying molecular mechanisms in skeletal muscle, bone, brain, heart, and adipose tissue. Furthermore, we explore the potential application of exercise in mitigating and managing related diseases, providing a theoretical framework for understanding the biological basis of exercise-induced health benefits and for developing targeted exercise interventions.

## 2. Overview of Angiogenesis

Angiogenesis is the biological process of forming new blood vessels from pre-existing vasculature, primarily through sprouting or intussusception, which serves as the foundation for embryonic development, tissue growth, and repair, and involves the proliferation, migration, and remodeling of vascular endothelial cells. In contrast, vasculogenesis refers to the de novo formation of blood vessels from endothelial progenitor or precursor cells and is classically associated with embryonic vascular development. Although these processes are biologically distinct, vasculogenic mechanisms may also contribute to vascular regeneration under certain physiological or pathological conditions [1,3]. Angiogenesis is tightly and dynamically regulated, in a spatially and temporally coordinated manner, by multiple signaling factors, including VEGF, Ang-1, Ang-2, and fibroblast growth factors (FGFs), among others [26].

Maintaining an appropriate angiogenic balance is essential for health. Insufficient angiogenesis can lead to decreased tissue perfusion, hypoxia, and impaired repair, contributing to the pathology of a range of conditions, including coronary heart disease, myocardial infarction, heart failure, and other ischemic cardiovascular diseases; sarcopenia, obesity, and related metabolic disorders; ischemic stroke; Alzheimer’s disease; and osteoporosis [2,3]. Conversely, excessive or abnormal angiogenesis promotes tumor growth, diabetic retinopathy, and other pathological conditions.

Therefore, in clinical practice, targeted “promotion of angiogenesis” has become an important strategy for treating ischemic and degenerative diseases. The primary goal is to stimulate the regeneration of functional capillaries and collateral arteries to restore tissue perfusion and blood supply and thereby improve organ function. A deep understanding of the regulatory mechanisms governing angiogenesis is therefore fundamental to developing effective therapeutic interventions.

## 3. Effect of Exercise on Angiogenesis in Skeletal Muscle

### 3.1. Effect of Exercise on Skeletal Muscle Angiogenesis and Its Underlying Mechanisms

Skeletal muscle, constituting approximately 40% of human body weight, is not only essential for locomotion but also plays a crucial role in cardiovascular and metabolic health. Moreover, skeletal muscle serves as an important source of various angiogenic factors, such as vascular endothelial growth factor-A (VEGF-A), Ang-1, and fibroblast growth factor-1 (FGF-1), whose production and release are upregulated in response to exercise or ischemic stimuli [27]. To maintain its function, skeletal muscle is endowed with an extensive capillary network [28]. The angiogenic response to exercise unfolds over two distinct temporal scales: acute molecular responses (e.g., rapid increases in VEGF mRNA and protein) and chronic structural adaptations (e.g., increased capillary density and capillary-to-fiber ratio). The sections that follow outline the major mechanistic pathways organized accordingly.

#### 3.1.1. Shear Stress and Endothelial Activation

During exercise, muscle contraction generates mechanical forces (e.g., shear stress, passive stretch) together with a marked increase in blood flow. These stimuli directly activate endothelial cell signaling and enhance the expression of angiogenic factors, thereby initiating capillary growth [29]. This endothelial activation can occur rapidly: a human study demonstrated that submaximal exercise effectively promotes an angiogenic response, showing a ninefold increase (*p* < 0.001) in VEGF messenger RNA (mRNA) expression in the skeletal muscle of the exercised leg after only three hours of bipedal kicking [30]. Thus, shear stress represents an acute mechanical trigger that precedes and sets the stage for subsequent structural remodeling.

#### 3.1.2. Hypoxia–HIF-1α Signaling

In parallel with mechanical activation, exercise-induced hypoxia engages the HIF-1α pathway, linking acute oxygen sensing to chronic adaptation. The elevated oxygen consumption in skeletal muscle during exercise can also lead to a decrease in local oxygen partial pressure, activating HIF-1α [5]. This molecular response can be distinguished from the slower structural changes it supports. Animal experiments have supported the role of HIF-1α in skeletal muscle angiogenesis. In a mouse model with conditional knockout of prolyl hydroxylase domain 2 (PHD2), which stabilizes HIF-1α, resting capillary density was elevated by approximately 40% in skeletal muscle. Following 4 weeks of treadmill training, the capillary-to-fiber (C/F) ratio increased in both genotypes; crucially, the training-induced increase in the C/F ratio and the improvement in running performance were significantly greater in PHD2 cKO mice than in wild-type controls, indicating that HIF-1α pathway activation synergizes with exercise training to enhance angiogenic adaptation rather than representing an absolute requirement [31]. Here, the acute stabilization of HIF-1α (molecular event) and the chronic rise in the C/F ratio (structural event) represent different biological readouts, consistent with the distinction emphasized throughout this review.

#### 3.1.3. VEGF and Angiopoietin Regulation

VEGF and the angiopoietin system are central molecular mediators that bridge acute signaling and chronic vessel remodeling. Exercise can rapidly and substantially upregulate the expression of multiple pro-angiogenic factors in skeletal muscle. Human studies have also found that the basal expression of VEGF protein in muscle increased approximately twofold after 10 days of single-leg knee extension training in healthy men—an intermediate-time-scale molecular change [32]. Long-term exercise also modulates the angiopoietin system. For example, 24 days of treadmill training in rats has been shown to induce a sustained increase in the ratio of Ang-1 to Ang-2 in skeletal muscle [33] (Table 1). Together, VEGF and angiopoietin regulation illustrate how transient factor-expression changes can support prolonged vascular remodeling, although changes in factor expression alone do not establish that new vessel formation has occurred.

#### 3.1.4. PGC-1α-Related Pathways

PGC-1α is a master regulator of mitochondrial biogenesis and oxidative metabolism and is proposed to link metabolic remodeling with angiogenic adaptation in skeletal muscle. Its role in angiogenesis is best understood as indirect and permissive rather than a direct effector mechanism. PGC-1α promotes the slow, oxidative fiber phenotype and can influence the expression of angiogenic factors such as VEGF and vascular endothelial growth factor receptor 2 (VEGFR2) in a fiber-type-dependent manner, thereby creating a metabolic environment that favors sustained capillary growth. In support of this coordinated relationship rather than direct causality, resistance exercise stimulates PGC-1α expression, which may induce IGF-1 and inhibit myostatin, promoting muscle hypertrophy—a structural adaptation that parallels the exercise-induced angiogenic response [34]. Similarly, short-term running exercise increases fibroblast growth factor-inducible 14 (Fn14) mRNA levels and is accompanied by an elevated proportion of oxidative muscle fibers and an increased capillary count [36]. Collectively, these findings suggest that PGC-1α-related pathways represent a plausible coordinating mechanism linking metabolic remodeling to capillary growth; however, direct evidence that PGC-1α itself drives angiogenesis independently of fiber-type and metabolic changes remains limited.

#### 3.1.5. Fiber-Type and Metabolic Remodeling

The chronic structural and phenotypic consequences of these pathways are reflected in fiber-type shifts and metabolic adaptation. Animal studies have shown that 8 weeks of voluntary wheel running (VWR) increased the proportion of type I muscle fibers, significantly enhanced capillary and lymphatic vessel density in the soleus muscle (SOL), and upregulated the expression of VEGF-C and VEGF-D [35]. This is considered an adaptive response to the VWR-induced shift toward a slower muscle phenotype and the associated increase in oxygen demand. Notably, the increase in type I fibers and capillary density after 8 weeks of VWR represents a chronic structural adaptation, distinct from the acute VEGF mRNA response described in Section 3.1.1. The precise morphological pattern of angiogenesis in skeletal muscle following exercise training nevertheless remains to be fully elucidated.

### 3.2. Exercise, Angiogenesis, and Mechanisms in Skeletal Muscle Diseases

#### 3.2.1. Sarcopenia

Sarcopenia is an age-related syndrome characterized by the progressive loss of skeletal muscle mass, strength, and function, leading to physical disability, loss of independence, and increased mortality [59]. The progressive muscle weakness and atrophy associated with aging are partly attributable to reduced capillary density. It is thought that the age-related decline in levels of angiogenic factors and the resultant microcirculatory insufficiency represent a common pathological mechanism [14]. A human study has indicated that exercise training counteracts this process by upregulating the PGC-1α/VEGF axis and enhancing endothelial nitric oxide signaling, thereby promoting skeletal muscle angiogenesis (manifested by increased capillarization) [60]. Furthermore, studies have shown that eight weeks of resistance training results in the acute upregulation of angiogenic markers such as VEGF and its receptor, vascular endothelial growth factor receptor 2 (VEGFR2), in skeletal muscle, concomitant with muscle hypertrophy and increased capillary density [61]. Animal experiments further support the notion, demonstrating that short-term running exercise significantly increases the mRNA levels of fibroblast growth Fn14 in the tibialis anterior and soleus muscles of mice, together with an elevated proportion of oxidative muscle fibers and an increased capillary count [36].

#### 3.2.2. Peripheral Arterial Disease (PAD)

Peripheral arterial disease (PAD), a common manifestation of atherosclerosis affecting over 200 million people worldwide, is primarily characterized by the stenosis or occlusion of lower limb arteries [62]. Promoting angiogenesis in ischemic limbs is a key therapeutic strategy for PAD [63]. Exercise-induced physiological hypoxia is a critical initiator of this process. The transient hypoxia resulting from high-intensity exercise activates oxygen-sensing mechanisms, stabilizing HIF-1α [64], which in turn upregulates growth factors such as VEGF to drive new blood vessel formation [65]. During exercise, various myokines released from skeletal muscle (including IL-6, CXCL family proteins, irisin, FSTL1, and IGF-1) jointly drive vascular regeneration by activating endothelial cells and recruiting precursor cells [66,67]. Animal studies have elucidated specific molecular mechanisms; for instance, acute running training can reduce the expression of FNIP1 in mouse skeletal muscle, and its specific deletion in muscle fibers can drive chemokine gene transcription via PGC-1α activation, thereby fostering angiogenesis [68]. Therefore, exercise not only serves as an effective preventive measure against PAD but also constitutes a vital component of its rehabilitation (Table 1).

## 4. Effect of Exercise on Bone Angiogenesis

### 4.1. Effect of Exercise on Bone Angiogenesis and Its Mechanism

Bone is a highly calcified and highly vascularized tissue. Its rich internal vasculature is essential for bone growth, repair, and homeostasis, supplying oxygen, nutrients, and growth factors and removing metabolic waste [69]. During bone development and remodeling, blood vessels not only provide nutritional support but also secrete angiocrine factors that help establish a local microenvironment conducive to the recruitment of osteoprogenitor cells and bone formation [70]. Consistent with this, numerous studies indicate that physical activity is beneficial for improving and maintaining bone quality [71].

VEGF is a master regulator of angiogenesis. Genetic studies in mice—for example, animals expressing only the VEGF120 isoform, which exhibits delayed vascular invasion during bone development—establish the general necessity of VEGF signaling for bone vascularization; however, such developmental evidence is distinct from exercise-induced effects and serves here only as background for the exercise studies that follow [72]. Turning to the exercise response, exercise can significantly enhance VEGF expression in bone [73]. Animal experiments demonstrate that exercise rapidly upregulates the VEGF system in bone: 10 days of treadmill training in 9-week-old male Wistar rats increased VEGF and VEGFR1 mRNA expression in the periosteum and epiphysis, and after two weeks of training the number of blood vessels in the proximal epiphyseal plate increased by 20%, despite there being no change in tibial bone mineral density [37]. Similarly, 8 weeks of swimming training enhanced bone capillary density in aged rats, an effect linked to the activation of both the angiopoietin and VEGF signaling pathways [38]. These observations support VEGF/angiopoietin-mediated angiogenesis in response to exercise in preclinical models.

Exercise-induced alterations in local hemodynamics and metabolism can create a relatively hypoxic microenvironment within bone, thereby activating the HIF-1α pathway. In bone biology, activation of the HIF-1α pathway is crucial because it directly couples angiogenesis with osteogenesis, primarily through VEGF produced by HIF-1α-overexpressing osteoblasts [41]. This coupling is further supported by bone-specific loading studies: in a rat forelimb compression model, the increase in periosteal blood vessels precedes the subsequent gain in bone area after fatigue loading, indicating that load-induced angiogenesis is spatially and temporally coordinated with bone formation [39]. The HIF-1α/VEGF axis therefore represents a plausible mechanistic link between mechanical loading and bone adaptation. Taken together, these findings indicate that exercise promotes angiogenesis–osteogenesis coupling in bone through the HIF-1α/VEGF axis (Table 1), with the caveat that the majority of this evidence derives from animal and mechanical-loading studies rather than human clinical trials.

### 4.2. Effect of Exercise on Angiogenesis in Bone Diseases and Its Mechanism

#### 4.2.1. Osteoporosis

Based on current evidence, exercise-induced angiogenesis is considered a plausible contributor to bone health, and the promotion of bone angiogenesis by exercise has been proposed as a mechanism underlying the beneficial effects of exercise on bone-related diseases. Osteoporosis is a systemic skeletal disorder characterized by low bone mass, deterioration of bone microarchitecture, increased bone fragility, and a consequent rise in fracture risk [74]. Notably, individuals with osteopenia face an elevated risk of subsequent fractures and premature mortality [75]. The various mechanical loads (such as tension, compression, and fluid shear stress) generated by exercise help reduce bone loss and enhance bone strength, serving as an effective non-pharmacological strategy to prevent age-related osteoporosis [15]. The extensive three-dimensional vascular network within bone is fundamental to its growth and metabolism, as angiogenesis and osteogenesis are closely coupled processes during both physiological development and repair [24]. A study using a rat forelimb compression model found that an increase in periosteal blood vessels precedes a corresponding increase in bone area following fatigue loading, indicating that the angiogenic–osteogenic response is spatially and temporally coordinated and can be adaptively regulated by the intensity of mechanical stimulation [39]. Clinical observations also support the link between vascular function and bone health; for instance, reduced arterial blood flow in the lower extremities is associated with increased bone loss in the hip and calcaneus of older women [76]. However, direct clinical or mediation evidence that angiogenesis itself is the causal mechanism through which exercise mitigates osteoporosis remains limited. Accordingly, the available data support a proposed rather than an established clinical mechanism.

#### 4.2.2. Bone Defect and Fracture Repair

The proposed core mechanism by which exercise promotes the healing of bone defects and fractures involves coupling angiogenesis with bone regeneration, primarily through activation of the HIF-1α/VEGF axis. In bone regeneration, HIF-1α plays a central regulatory role and is critical for constructing the vascular network required for repair [77]. In processes such as distraction osteogenesis, the upregulation of VEGF expression regulated by HIF-1α transcription is key to initiating vascular infiltration and subsequently promoting bone regeneration [78]. Animal model studies have confirmed that inhibiting VEGF activity disrupts the repair of femoral fractures and cortical bone defects in mice, while VEGF drives the vascularization, ossification, and maturation of fracture calluses by promoting angiogenesis [79]. Direct evidence demonstrates that exercise itself accelerates this healing process: mice with access to running wheels for voluntary exercise showed faster healing of calvarial defects compared to sedentary controls. This accelerated repair was accompanied by more newly formed woven bone and the generation of new, highly vascularized periosteum [40], suggesting that exercise can stimulate angiogenesis during bone repair in preclinical models (Table 1).

It is important to distinguish these repair/fracture-healing models from the chronic, age-related bone loss of osteoporosis. The evidence above derives largely from controlled bone defect, distraction osteogenesis, and fracture repair settings in animals—active regenerative contexts that differ from clinical osteoporosis. Therefore, while they support a role for exercise in angiogenesis during bone repair, they do not directly establish that exercise-induced angiogenesis prevents or treats osteoporosis in humans; such extrapolation requires dedicated clinical studies. Moreover, the angiogenic–osteogenic coupling described here is beneficial in the context of bone repair and maintenance; however, uncontrolled angiogenesis in conjunction with inflammation may contribute to osteolytic pathologies in certain settings.

## 5. Effect of Exercise on Cerebral Angiogenesis

### 5.1. Effect of Exercise on Brain Angiogenesis and Its Mechanism

As a high-energy-demand organ with limited intrinsic energy stores, the brain is critically dependent on a continuous and stable blood supply to sustain its complex cognitive functions and homeostasis [80]. Cerebral blood flow is precisely regulated to dynamically redistribute blood to the most active brain regions, ensuring a close match between oxygen/nutrient delivery and local metabolic demand [81]. Long-term physical exercise induces adaptive changes in the brain, including the promotion of capillary neogenesis in regions such as the cortex and hippocampus and the enhancement of local cerebral blood flow under conditions of neural activation [82].

VEGF is a central mediator of exercise-induced cerebral angiogenesis. Animal studies have shown that 1 week of voluntary wheel running upregulates VEGF-A protein expression in the mouse hippocampus and promotes angiogenesis, an effect that can be inhibited by VEGF blockers [43]. Beyond VEGF, exercise modulates other key factors. BDNF has been identified as a key molecule coupling exercise-induced neurogenesis with angiogenesis [83]. Human studies confirm that acute exercise (e.g., 4 h rowing) can elevate circulating levels of brain-derived neurotrophic factor (BDNF) by 2- to 3-fold above the resting state. It is important to note that this observation, being a peripheral/circulating measurement in humans, does not by itself demonstrate cerebral angiogenesis in humans or establish a BDNF-mediated angiogenic mechanism in the human brain. Animal experiments further revealed that exercise increases BDNF mRNA expression in the hippocampus and cortex by 3- to 5-fold, providing direct evidence of central BDNF upregulation in a preclinical model [42]. Thus, while human and animal findings are mechanistically coherent, the clinical relevance and direct demonstration of exercise-induced cerebral angiogenesis in humans remain to be established.

Lactate, produced during exercise, functions not merely as a metabolic byproduct but also as an important signaling molecule. Lactate can activate its receptor, hydroxycarboxylic acid receptor 1 (HCAR1), to upregulate VEGF expression and thereby promote angiogenesis [43]. Furthermore, exercise improves vascular endothelial function. An 8-week treadmill training study in aged rats demonstrated that exercise upregulated the expression of both VEGF and eNOS in the brain, leading to a significant increase in regional cerebral blood flow and capillary density. Nitric oxide (NO), produced by eNOS, is a key mediator for maintaining vascular tone and supporting angiogenesis. Importantly, exercise-induced vascular adaptation involves not only an increase in vessel number but also structural remodeling. Animal experiments showed that 5 weeks of aerobic exercise increased the number of capillary sprouts and enlarged vessel diameter in the cerebral motor cortex of mice [44], underscoring that exercise effectively promotes both angiogenesis and vascular remodeling in the cerebral motor cortex (Table 1). Notably, the structural changes described here (capillary sprouts, enlarged diameter) provide direct evidence of vascular remodeling, whereas the concurrent increase in regional cerebral blood flow reflects a distinct functional outcome.

### 5.2. Effect of Exercise on Angiogenesis in Brain Diseases and Its Mechanism

#### 5.2.1. Alzheimer’s Disease (AD)

Exercise mitigates cerebrovascular dysfunction, a key pathological feature of Alzheimer’s disease (AD), through mechanisms that include promoting angiogenesis and improving cerebral blood flow. These two outcomes should be distinguished: angiogenesis denotes structural vessel growth, whereas increased cerebral blood flow reflects functional perfusion. The mechanisms are multifaceted. First, the increased shear stress from enhanced blood flow during exercise boosts vascular eNOS activity and NO bioavailability while also upregulating VEGF expression [16]. Second, exercise-induced lactate acts as a signaling molecule, activating its receptor HCAR1 to enhance the expression of BDNF and VEGF, thereby synergistically improving cerebral perfusion [84]. However, whether these vascular changes translate into cognitive benefit remains to be established. Third, both long-term aerobic exercise and high-intensity interval training can broadly elevate the levels of VEGF, NO, and other pro-angiogenic factors in the brain, promoting overall vascular health [85]. Animal studies confirm that exercise training effectively upregulates the VEGF system and increases microvessel density in the aged brain, thereby providing direct evidence of angiogenesis in a preclinical AD-relevant context [86]. However, whether this structural angiogenic response mediates the clinical benefits of exercise in human AD remains to be established.

#### 5.2.2. Vascular Dementia (VD)

Vascular dementia (VD), primarily caused by cerebrovascular injury, is the second most common cause of dementia in the elderly [87]. Exercise may prevent and treat VD by improving cerebrovascular function. Animal studies demonstrate that 4 weeks of voluntary wheel running improves cortical cerebral blood flow via eNOS-mediated vasodilation while also promoting angiogenesis (increased microvessel density) [45]. Another study showed that 5 weeks of running training significantly increased the diameter and density of hippocampal microvessels in mice, a mechanism linked to the activation of the lactate receptor HCAR1 and its downstream ERK1/2-PI3K/Akt pathway [46]. Furthermore, 4 weeks of running training promoted the expression of pro-angiogenic factors VEGF and FGF-2, increased cortical capillary length, volume, and surface area, and reduced the expression of the anti-angiogenic factor endostatin [88,89]. Here, increases in microvessel diameter and density represent structural angiogenic outcomes, while the accompanying improvements in cortical cerebral blood flow are functional vascular responses; both are observed in animal models, but their causal contribution to cognitive outcomes in human VD remains to be directly tested.

#### 5.2.3. Ischemic Stroke

Ischemic stroke, a leading global cause of death and disability, results from cerebral vascular occlusion. Post-stroke rehabilitation exercise is crucial for promoting functional recovery [90]. Studies show that one week of moderate or high-intensity running training in rats after stroke significantly increases the expression of VEGF, its receptor VEGFR2, and BDNF in the ischemic region [47]. Treadmill exercise also increases microvessel density by upregulating VEGF and laminin, thereby promoting neurological recovery [48]. The underlying molecular mechanism involves coordinated crosstalk among multiple pathways. Exercise enhances HIF-1α expression, promoting the transcription of downstream pro-angiogenic genes to drive angiogenesis. In parallel, exercise activates the BDNF/TrkB/CREB signaling cascade, which may synergistically facilitate angiogenesis and neurogenesis—a distinct cellular process that may be coupled to angiogenesis. Thus, while angiogenesis and neurogenesis can occur in parallel and may share upstream signals (e.g., VEGF, BDNF), they represent different biological endpoints. Additionally, exercise increases the expression of membrane-type 1 matrix metalloproteinase (MT1-MMP), a key enzyme for extracellular matrix remodeling that creates a permissive microenvironment for new vessels [49,50,51,52]. These multi-target effects collectively drive angiogenesis and functional remodeling in the ischemic penumbra (Table 1). The evidence for angiogenesis per se derives from measures such as increased microvessel density and VEGF/laminin upregulation, whereas neurological recovery is a downstream functional outcome whose mediation by angiogenesis requires further validation. In addition, while post-stroke angiogenesis may support tissue repair, the quality and maturity of newly formed vessels are critical determinants of functional recovery; immature vasculature may be associated with hemorrhagic transformation or exacerbated edema.

## 6. Effects of Exercise on Cardiac Angiogenesis

### 6.1. Effects of Exercise on Cardiac Angiogenesis and Its Mechanism

Cardiovascular disease remains the leading cause of death worldwide, posing a severe threat to global health [17]. Exercise confers cardioprotection through an integrated program of adaptation, of which adaptive remodeling of the myocardial vascular network is a key component. Beyond angiogenesis, exercise is well established to elicit improvements in endothelial function, autonomic regulation, mitochondrial biogenesis and remodeling, and hemodynamic loading patterns [91,92,93]; this broader adaptive context is acknowledged here, although the present section focuses on the angiogenic component. In healthy, exercise-trained hearts, unlike the capillary rarefaction characteristic of pathological myocardial hypertrophy, exercise-induced physiological myocardial hypertrophy is typically accompanied by a proportionate or even greater increase in capillary density, ensuring adequate blood supply to the enlarged cardiomyocytes [94,95]. Exercise enhances coronary vasodilation, improves myocardial perfusion, and expands the capillary network by promoting new blood vessel formation and collateral circulation [96]. Thus, angiogenesis is one important feature of a broader physiological remodeling response in the healthy heart.

In physiological, exercise-trained healthy models, research indicates that different exercise modalities converge on promoting cardiac angiogenesis through distinct yet complementary molecular pathways. A study showed that 8 weeks of weight-bearing climbing resistance training upregulated VEGF and irisin expression and promoted angiogenesis in the mouse heart, suggesting a multifactorial mechanism [97]. In a swimming training model, 8 weeks of exercise promoted myocardial angiogenesis during adaptive remodeling by activating the Tescalcin (TESC)-mediated C-SRC/insulin-like growth factor 1 receptor β (IGF1Rβ)/PI3K/Akt/VEGF-A signaling pathway [98]. Long-term aerobic exercise is also highly effective. Following 8 weeks of treadmill training in rats, the cardiac expression of numerous angiogenesis-related factors—including eNOS, VEGF, fetal liver kinase-1 (Flk-1), HIF-1α, PGC-1α, and Ang-1—was upregulated, collectively inducing myocardial angiogenesis and improving function [53]. Another 12-week treadmill training study confirmed that exercise significantly increased myocardial protein levels of HIF-1α, PGC-1α, VEGF, and the endothelial progenitor cell marker CD34+, working in concert to promote comprehensive cardiac adaptation in which angiogenesis is one component [99] (Table 1).

### 6.2. Effects of Exercise on Angiogenesis in Heart Disease and Its Mechanism

The evidence described below concerns pathological cardiac settings, which differ importantly from the physiological angiogenesis in healthy trained hearts discussed in Section 6.1. In diabetic cardiomyopathy and myocardial infarction (MI), angiogenic responses occur against a background of metabolic stress, ischemia, or tissue remodeling, and the mechanisms and therapeutic implications may not be directly equivalent to those in healthy models.

#### 6.2.1. Diabetic Heart Disease

In pathological settings, diabetes mellitus is a common chronic metabolic disease associated with a significantly increased risk of cardiovascular complications [100]. Exercise can ameliorate the impaired cardiac angiogenesis characteristic of the diabetic state. In streptozotocin-induced type I diabetic rats, just 5 days of treadmill exercise reversed the diabetes-induced downregulation of cardiac VEGF expression and promoted vascular endothelial expansion [101]. In a type 2 diabetes model, 8 weeks of exercise training significantly upregulated the expression of VEGF-A, its receptor VEGFR2, and downstream ERK1/2 and Akt signaling molecules in myocardial tissue, thereby activating the pro-angiogenic pathway [54,55]. Additionally, 6 weeks of voluntary wheel running increased cardiac expression of VEGF-A and stromal cell-derived factor-1α (SDF-1α) in type I diabetic rats, which may promote angiogenesis through complementary pathways [102,103]. Further studies demonstrated that 8 weeks of treadmill exercise increases levels of the pro-angiogenic factors VEGF and matrix metalloproteinase-2 (MMP-2) in the hearts of type 2 diabetic rats, while reducing expression of the anti-angiogenic factor transforming growth factor-β (TGF-β) and its inhibitor TIMP-2, thereby improving angiogenesis and attenuating cardiac fibrosis [104]. These findings, predominantly from streptozotocin-induced type 1 and type 2 diabetic rat models, demonstrate that exercise can restore or augment angiogenic signaling in the diabetic heart; whether equivalent effects translate to human diabetic cardiomyopathy remains to be established.

MicroRNAs (miRNAs) are critical regulators of angiogenesis, and exercise exerts therapeutic effects partly by modulating specific miRNAs [105]. For instance, 6 weeks of treadmill training downregulated cardiac miR-34a expression in type 2 diabetic rats while upregulating protein levels of SIRT1 and HIF-1α, promoting angiogenesis [106]. Similarly, 6 weeks of training upregulated the diabetic rats’ cardiac miR-126 and RAF protein expression while downregulating the target protein SPRED-1, synergistically enhancing the pro-angiogenic response [56]. Other studies show that 6 weeks of exercise elevates cardiac miR-132 levels in diabetic rats, further contributing to angiogenesis [107]. Thus, the miRNA-mediated regulation of angiogenesis described here represents a plausible mechanistic layer in preclinical diabetic-heart models, complementary to but distinct from the physiological adaptation observed in healthy trained hearts.

#### 6.2.2. Myocardial Infarction (MI)

Myocardial infarction leads to sustained ischemia and hypoxia, and promoting angiogenesis is key to cardiac repair [108]. In the MI setting—an active reparative context distinct from physiological training—both high-intensity interval training and moderate-intensity continuous training, sustained for 4–8 weeks, can promote post-infarction angiogenesis and may positively impact cardiac repair in preclinical models [109]. As early as 3 days after MI, aerobic exercise upregulated myocardial expression of VEGF and its receptors Flt-1 and Flk-1 at both mRNA and protein levels, increasing microvessel density and reducing infarct size in MI mice [110]. A 4-week aerobic exercise intervention significantly upregulated cardiac fibroblast growth factor 21 (FGF21) expression, promoting angiogenesis through activation of the PI3K/Akt/VEGF signaling pathway in mice [58]. Four weeks of resistance training, aerobic training, or combined training promoted myocardial angiogenesis and reduced infarct size by enhancing bone marrow-derived endothelial progenitor cell (EPC) function and activating the neuregulin-1 (NRG-1) pathway in MI mice [111]. Studies also found that both aerobic and resistance exercise stimulate the secretion of follistatin-like protein 1 (FSTL1) from the heart and skeletal muscle, promoting post-MI cardiac angiogenesis and repair through the DIP2A–Smad2/3 signaling pathway in rats [112]. MiRNAs also serve as important regulators of post-MI angiogenesis by targeting VEGF, PI3K, eNOS, and related pathways [113]. In MI rats, 4 weeks of treadmill training increased HIF-1α and miR-126 expression in the heart while decreasing the expression of their target genes PIK3R2 and SPRED1, promoting angiogenesis via activation of the PI3K/Akt/eNOS and MAPK pathways [57]. Another study found that combined aerobic and resistance training improved rats’ cardiac angiogenesis and function after MI by inhibiting miR-15a and miR-146a and upregulating the VEGF/PI3K/eNOS signaling pathway [114] (Table 1). In MI, the angiogenic response occurs against a background of ischemia, infarction, and tissue remodeling, and the observed benefits reflect the integration of multiple pathways rather than angiogenesis alone. Accordingly, while exercise-induced angiogenesis is a consistent finding across these disease models, it should not be regarded as the single central or causal mediator of exercise benefit in humans; the relative contributions of angiogenesis, endothelial function, autonomic regulation, mitochondrial remodeling, and hemodynamic adaptation remain to be disentangled in well-designed clinical studies. Furthermore, although exercise-induced angiogenesis after MI is generally reparative, the balance between productive revascularization and the formation of immature, leaky microvessels remains a key consideration for clinical translation.

## 7. Effects of Exercise on Adipose Tissue Angiogenesis and Its Mechanism

Obesity represents a major public health challenge. The escalating prevalence of overweight and obesity in recent decades has imposed a significant medical and socioeconomic burden [115]. During the development of obesity and aging, adipose tissue often undergoes functional deterioration, accompanied by a reduction in vessel density and impaired angiogenesis, a state described as “vascular rarefaction” [116]. Physical exercise, including both resistance and endurance training, is a highly effective intervention for preventing and managing obesity and its metabolic complications, exerting multiple beneficial effects on adipose tissue morphology and function [117,118,119].

A key mechanism through which exercise improves adipose tissue health is by promoting angiogenesis. This enhances tissue perfusion, alleviates hypoxia, and boosts metabolic activity. Human studies have found that regular moderate-intensity exercise (at least four times per week) in adults with obesity upregulates the expression of VEGF-A and the platelet endothelial cell adhesion molecule-1 (CD31) in adipose tissue, leading to a significant increase in capillary number and density [18]. Animal experiments provide further mechanistic insight. One study revealed that a 7-week exercise intervention ameliorated adipose tissue vascular rarefaction in high-fat diet-induced obese mice, a process linked to exercise-induced activation of the mouse double minute 2 homolog (MDM2) and subsequent inhibition of forkhead box protein O1/3a (FoxO1/3a) expression [19]. Another study demonstrated that 8 weeks of treadmill training increased VEGF-A expression, promoted angiogenesis, and reduced lactate levels in the adipose tissue of obese mice, indicating that exercise effectively alleviates adipose tissue hypoxia [20].

However, the magnitude and nature of these angiogenic responses are influenced by adipose depot, disease stage, and inflammatory context. Adipose tissue is heterogeneous, and angiogenic responses to exercise are likely depot-specific. Subcutaneous (sWAT) and visceral (vWAT) white adipose tissue depots differ in their baseline angiogenic capacity—subcutaneous fat exhibits a greater capacity for capillary expansion than visceral fat, although this capacity declines with morbid obesity [120]—while brown adipose tissue (BAT) responds to thermogenic cues with distinct VEGF-driven vascular expansion [121]. Consequently, the mechanisms and metabolic consequences of exercise-induced angiogenesis may differ between depots, and findings in one depot should not be directly extrapolated to another.

Furthermore, adipose angiogenesis is context-dependent. During healthy adipose expansion, angiogenesis is coordinated with adipogenesis, supporting tissue growth and maintaining perfusion; in contrast, in established obesity, inadequate vascularization leads to capillary rarefaction and pathological vascular remodeling—including endothelial dysfunction, fibrosis, and defective vessel maturation—that impairs adipose function [122,123]. Adipose tissue angiogenesis can therefore have either beneficial or detrimental consequences for tissue function depending on the context and obesity status [124,125]. Exercise appears most beneficial when it restores or augments a healthy, well-organized adipose vasculature, rather than promoting angiogenesis under all circumstances. The hypoxia–angiogenesis axis is also closely intertwined with inflammation: in obese adipose tissue, hypoxia stabilizes HIF-1α, which upregulates both pro-angiogenic factors (e.g., VEGF) and pro-inflammatory adipokines (e.g., IL-6, TNF-α, MCP-1) [126], such that exercise-induced improvements in perfusion and capillary density may alleviate hypoxia and, secondarily, dampen hypoxia-driven inflammation—although direct evidence that angiogenesis itself mediates this anti-inflammatory effect remains limited [127] (Table 1).

## 8. Limitations and Critical Synthesis

Although this review has emphasized the adaptive, beneficial consequences of exercise-induced angiogenesis, it is important to recognize that angiogenesis is not invariably advantageous. The health impact of new vessel formation depends critically on the tissue context, disease state, vascular maturity, inflammatory microenvironment, cancer biology, and metabolic status—the same variables that determine whether a vascular response supports repair or contributes to pathology. This nuance is particularly relevant to the earlier observation that excessive or abnormal angiogenesis underlies tumor growth and diabetic retinopathy. In a malignant tumor, exercise-induced upregulation of VEGF and other pro-angiogenic factors could, in principle, support pathological neovascularization and provide routes for metastatic dissemination, although moderate voluntary exercise may instead improve tumor perfusion and reduce hypoxia, thereby favoring vascular normalization rather than unchecked vessel growth [128]. Likewise, proliferative diabetic retinopathy is driven by hyperglycemia- and ischemia-induced retinal neovascularization that is structurally defective and clinically detrimental—fundamentally distinct from the controlled angiogenesis observed in skeletal muscle or the heart [129]. A key concept is therefore that vascular quantity does not necessarily equate to vascular quality: newly formed capillaries must acquire pericyte coverage, a basement membrane, and intact endothelial junctions to become stable and barrier-competent [128]. When endothelial sprouting outpaces this maturation process, the resulting immature, leaky vasculature may worsen edema and inflammation rather than improve perfusion. Context also matters in adipose tissue, where angiogenesis that is adaptive under healthy expansion may become maladaptive in established obesity, perpetuating a dysfunctional, inflammatory microenvironment. Accordingly, exercise-induced angiogenesis should not be viewed as an unequivocally beneficial process, and future studies should assess not only the extent of vessel formation but also vessel architecture, perfusion, permeability, and mural-cell coverage across disease contexts.

Beyond this context dependency, several additional limitations warrant consideration in this review. First, angiogenesis is operationalized by a range of endpoints that capture different biological phenomena. Changes in the expression of pro-angiogenic factors such as VEGF, HIF-1α, BDNF, or eNOS reflect molecular signaling activity, whereas increased capillary density, structural vascular remodeling, and the formation of functional new vessels represent distinct anatomical and physiological readouts. These endpoints are often reported interchangeably, yet they do not carry the same evidential weight. In particular, changes in tissue perfusion, endothelial function, metabolism, inflammation, or training adaptation may coincide with angiogenesis without being caused by it. Second, there is considerable heterogeneity across studies in terms of exercise modality, intensity, duration, species, age, disease status, tissue sampled, and the specific outcome used to index angiogenesis. This diversity limits the extent to which findings can be directly compared or generalized across tissues and experimental systems. Third, the evidence base is predominantly preclinical. Much of the mechanistic insight—especially for brain and bone—comes from animal and cellular studies, with relatively limited causal evidence from human exercise interventions. As a result, the extent to which these findings translate to human physiology and disease remains uncertain. Finally, a causal role of angiogenesis in clinical outcomes has not been firmly established. Most studies demonstrate associations or support plausible mechanisms, but few provide formal mediation evidence that angiogenesis itself drives the observed health benefits. Therefore, the clinical implications discussed in this review should be interpreted as hypotheses to be tested in well-designed human studies. Despite these limitations, the convergence of evidence across multiple tissues and model systems supports the broader conclusion that exercise elicits tissue-specific angiogenic responses with potential translational relevance.

## 9. Conclusions and Prospects

**Figure 1:**
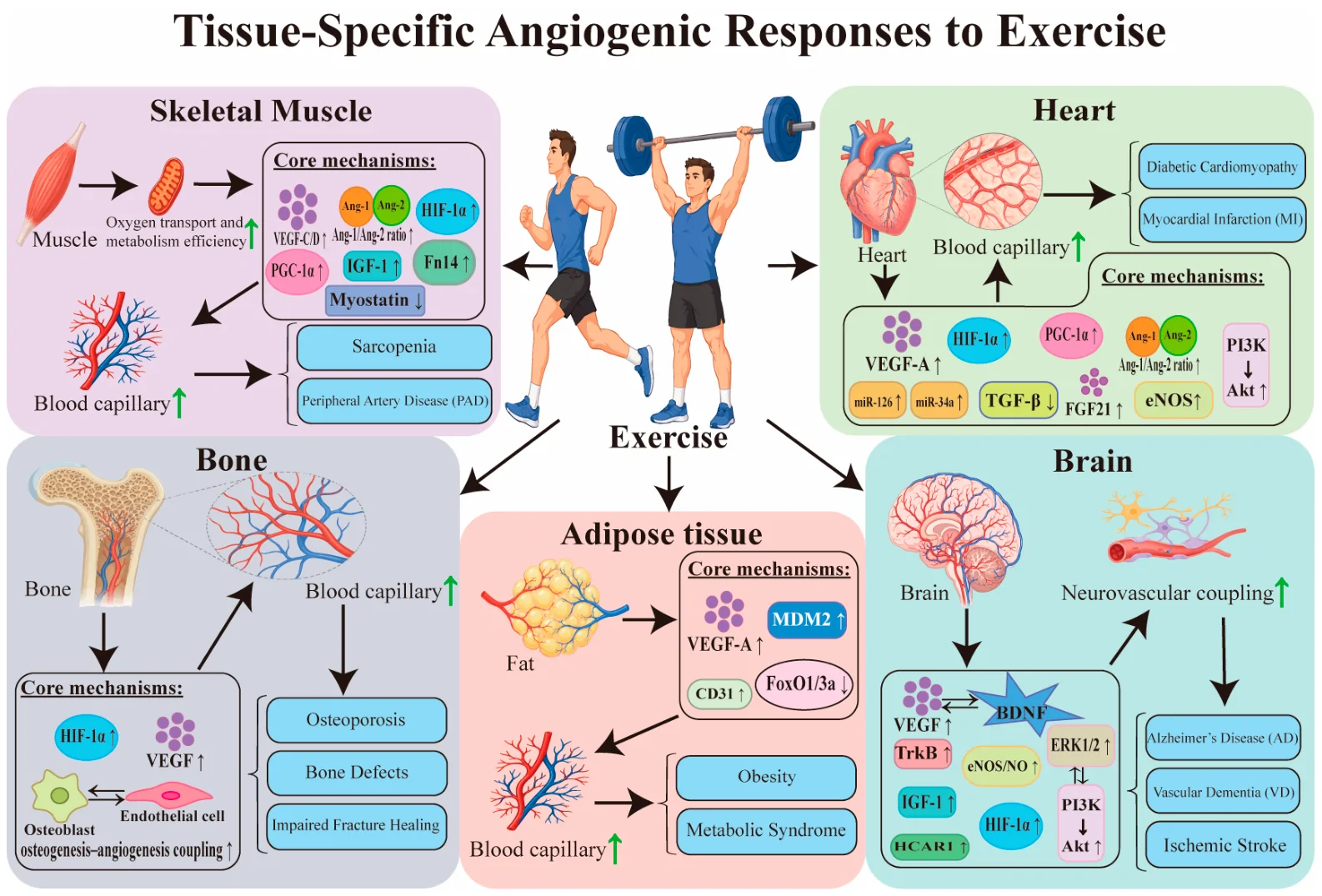
Comparative overview of tissue-specific angiogenic adaptations induced by exercise. The directional arrows summarize proposed or experimentally supported relationships derived from different evidence models; not all pathways have been causally demonstrated in humans. Exercise promotes distinct angiogenic responses across different tissues and organs (↑, increase or activate; ↓, reduce or inhibit). (1) In skeletal muscle (human + animal), angiogenesis mainly supports oxygen delivery and metabolic adaptation through upregulation of VEGF-C/D, Ang-1/2, PGC-1α, HIF-1α, IGF-1, and Fn14, accompanied by myostatin suppression. (2) In bone (animal + cell), exercise-induced angiogenesis is closely coupled with osteogenesis via VEGF/HIF-1α and FGF-2-related pathways, facilitating bone remodeling and repair. (3) In the brain (animal), increased VEGF, BDNF, IGF-1, HIF-1α, ERK1/2-PI3K/Akt, and eNOS enhance neurovascular coupling and cerebral perfusion, contributing to neuroprotection and cognitive improvement. (4) In the heart (animal), exercise activates VEGF-A, PI3K/Akt, Ang-1/2, PGC-1α, FGF-21, and eNOS-associated pathways to improve myocardial perfusion and ischemic repair while inhibiting pro-fibrotic signaling such as TGF-β. (5) In adipose tissue (human + animal), exercise promotes vascular remodeling and metabolic homeostasis through VEGF-A and MDM2 upregulation and FoxO1/3a reduction and increases capillary density. Overall, exercise-induced angiogenesis exhibits marked tissue specificity and may play a crucial role in mitigating and managing musculoskeletal, cardiovascular, metabolic, and neurodegenerative diseases.

This review summarizes the tissue-specific effects of exercise in promoting angiogenesis across key organs and the underlying molecular mechanisms. Accumulating evidence indicates that regular exercise serves as a potent physiological stimulus that precisely initiates and regulates angiogenic programs by integrating signals from mechanical stress, metabolic shifts, hypoxic stress, and inflammatory modulation. Crucially, these effects exhibit remarkable tissue specificity. In skeletal muscle, exercise primarily induces capillary proliferation by upregulating VEGF, activating the HIF-1α/PGC-1α pathway, and modulating the Ang-1/Ang-2 ratio, which is associated with meeting increased metabolic demands and counteracting pathological vascular decline in conditions like sarcopenia and peripheral artery disease. Within the skeletal system, exercise couples angiogenesis with osteogenesis via the HIF-1α/VEGF and FGF-2 signaling axes, which may contribute to maintaining bone mineral density, promoting fracture healing, and mitigating osteoporosis. In the brain, exercise-induced angiogenesis is closely coupled to neuroplasticity. Coordinated actions of the BDNF, VEGF, and lactate–HCAR1 signaling pathways increase capillary density and cerebral blood flow in regions such as the hippocampus and cortex, providing a vascular foundation that may support the cognitive benefits of exercise, delay the progression of Alzheimer’s and vascular dementia, and facilitate post-stroke recovery. In the heart, exercise promotes myocardial angiogenesis and coronary collateral circulation by activating pathways such as PI3K/Akt/VEGF and upregulating factors such as irisin and FSTL1, which is associated with enhancing cardiac function, ameliorating ischemia, and supporting post-infarction remodeling. In adipose tissue, exercise counteracts obesity-related “vascular rarefaction” by upregulating VEGF-A and improving the hypoxic microenvironment, thereby increasing capillary density and potentially promoting metabolic health (Figure 1). It should be emphasized that most of the evidence reviewed herein is mechanistic and preclinical in nature; direct evidence that angiogenesis itself mediates clinically meaningful disease outcomes in humans remains limited for several tissues.

Despite significant progress, future research should prioritize several directions. First, comprehensive comparisons are needed to delineate the differential impacts of various exercise modalities (e.g., endurance, resistance, and high-intensity interval training), as well as the parameters of intensity, frequency, and duration, on angiogenesis in specific tissues. Critically, studies should standardize the timing of tissue sampling relative to the last exercise bout, since angiogenic signals and endothelial responses are highly time-dependent and comparisons across studies are currently confounded by heterogeneous sampling windows. Developing personalized, precise exercise prescriptions that consider an individual’s age, sex, genotype, and disease status to maximize vascular benefits is a key translational goal; such prescriptions must be informed by comparative studies of metabolic disease versus healthy tissue, as the angiogenic response—and its dependence on exercise modality and intensity—may differ fundamentally between diseased and non-diseased states. However, this prospect remains to be validated through well-designed human intervention studies and clinical mediation trials. The application of cutting-edge technologies such as single-cell sequencing, spatial transcriptomics, and proteomics will be vital to identify exercise-responsive endothelial cell subtypes and map their dynamic changes and microenvironment interactions before and after exercise, facilitating the discovery of novel regulatory targets. Finally, the roles of the gut microbiota, its metabolites, and tissue-specific myokines/adipokines as mediators of exercise’s systemic effects are gaining prominence. Understanding how these factors mediate exercise-induced angiogenesis in distal tissues (e.g., the brain, adipose tissue) represents a promising and emerging frontier in the field.

## Figures and Tables

**Table 1:** Comparative landscape of tissue-specific angiogenic responses in physiological and disease contexts.

Organ Tissue	Primary Physiological Objectives	Key Characteristic Pathways/Factors	Human Evidence	Animal Evidence	Vitro/Mechanistic Evidence	Related Diseases	Overall Evidence Strength
Skeletal Muscle	Improve oxygen delivery and metabolic efficiency; enhance endurance and muscle fiber adaptation	VEGF ↑; VEGF-C/D ↑; Ang-1/Ang-2 ratio ↑; HIF-1α ↑; PGC-1α ↑; IGF-1 ↑; Fn14 ↑; myostatin ↓	VEGF mRNA ↑ 9-fold after acute exercise [30]; VEGF protein ↑ 2-fold after 10-day training [32]; PGC-1α4 ↑; Myostatin ↓ after resistance exercise [34].	VEGF-C/D ↑ and capillary/lymphatic density ↑ [35]; HIF-1α activation and capillary density ↑ [31]; Ang-1/Ang-2 ratio ↑ after treadmill training [33]; Fn14 ↑ and capillary number ↑ after running [36].	—	Sarcopenia; peripheral artery disease (PAD)	Moderate
Bone	Couple angiogenesis with osteogenesis; maintain bone density and repair capacity	HIF-1 α/VEGF axis ↑; osteogenesis–angiogenesis coupling ↑	—	VEGF/VEGFR1 ↑ and bone vessel number ↑ after treadmill exercise [37]; bone capillary density ↑ after swimming [38]; mechanical loading promotes angiogenesis–osteogenesis coupling [39]; exercise-enhanced angiogenesis during bone-defect healing [40].	HIF-1α ↑ and VEGF ↑ [41]	Osteoporosis; bone defects; impaired fracture healing	Limited
Brain	Improve cerebral blood flow and neurovascular coupling; promote cognition and neuroplasticity	BDNF-VEGF/TrkB coupling ↑; lactate–HCAR1 signaling axis ↑; eNOS/NO pathway ↑; HIF-1α ↑; IGF-1 ↑; ERK1/2-PI3K/Akt ↑	BDNF ↑ [42]	VEGF ↑ and cerebral angiogenic adaptation [43,44]; eNOS ↑ with neovascularization/cerebral blood flow ↑ [45]; lactate/HCAR1 promotes VEGF-A ↑ and angiogenesis ↑ [46]; HIF-1α and BDNF/TrkB signaling contribute to post-stroke angiogenesis [47,48,49,50,51,52].	—	Alzheimer’s disease (AD); vascular dementia (VD); ischemic stroke	Limited
Heart	Enhance myocardial perfusion and collateral circulation; promote post-ischemic repair	HIF-1 α, PGC-1 α↑ pathway; VEGF-A, Ang-1/Ang-2 ↑ ratio regulation; PI3K/Akt ↑; miR-126 ↑, miR-34a ↓; TGF-β ↓; FGF21 ↑; eNOS ↑	—	HIF-1α/PGC-1α ↑; VEGF/eNOS/Ang-1 ↑ [53]. VEGF-A/PI3K/Akt signaling ↑ [54,55]; miR-126 ↑ and PI3K/Akt/eNOS signaling ↑ [56,57]; miR-34a ↓ and HIF-1α ↑; FGF21 ↑ and PI3K/Akt/VEGF signaling ↑ [58].	—	Diabetic cardiomyopathy; myocardial infarction (MI)	Limited
Adipose Tissue	Improve adipose tissue perfusion and metabolic microenvironment; alleviate hypoxia and inflammation	VEGF-A ↑; MDM2 ↑; CD31 ↑; FoxO1/3a ↓	VEGF-A mRNA ↑ after acute aerobic exercise. CD31 mRNA ↑ in regularly exercising adults [18].	MDM2 ↑ and FoxO1/3a ↓ with adipose vascular regrowth [19]; VEGF-A ↑ and adipose angiogenesis ↑ after treadmill training [20].	—	Obesity; metabolic syndrome	Moderate

Strength of evidence: moderate = human and preclinical support; limited = preclinical evidence only. ↑, increase or activate; ↓, reduce or inhibit.

## Data Availability

No new data were created or analyzed in this study. Data sharing is not applicable to this article.

## References

[1] Potente M., Gerhardt H., Carmeliet P. (2011). Basic and Therapeutic Aspects of Angiogenesis. Cell.

[2] Carmeliet P. (2005). Angiogenesis in Life, Disease and Medicine. Nature.

[3] Carmeliet P., Jain R.K. (2011). Molecular Mechanisms and Clinical Applications of Angiogenesis. Nature.

[4] Pedersen B.K., Saltin B. (2015). Exercise as Medicine—Evidence for Prescribing Exercise as Therapy in 26 Different Chronic Diseases. Scand. J. Med. Sci. Sports.

[5] Lindholm M.E., Rundqvist H. (2016). Skeletal Muscle Hypoxia-Inducible Factor-1 and Exercise. Exp. Physiol..

[6] Gorski T., De Bock K. (2019). Metabolic Regulation of Exercise-Induced Angiogenesis. Vasc. Biol..

[7] Lou J., Wu J., Feng M., Dang X., Wu G., Yang H., Wang Y., Li J., Zhao Y., Shi C. (2022). Exercise Promotes Angiogenesis by Enhancing Endothelial Cell Fatty Acid Utilization via Liver-Derived Extracellular Vesicle miR-122-5p. J. Sport. Health Sci..

[8] Bao C., Yang Z., Li Q., Cai Q., Li H., Shu B. (2020). Aerobic Endurance Exercise Ameliorates Renal Vascular Sclerosis in Aged Mice by Regulating PI3K/AKT/mTOR Signaling Pathway. DNA Cell Biol..

[9] Colombo R., Siqueira R., Conzatti A., de Lima Seolin B.G., Fernandes T.R.G., Godoy A.E.G., Litvin I.E., Silva J.M.A., Tucci P.J.F., da Rosa Araújo A.S. (2016). Exercise Training Contributes to H2O2/VEGF Signaling in the Lung of Rats with Monocrotaline-Induced Pulmonary Hypertension. Vasc. Pharmacol..

[10] Simmons G.H., Wong B.J., Holowatz L.A., Kenney W.L. (2011). Changes in the Control of Skin Blood Flow with Exercise Training: Where Do Cutaneous Vascular Adaptations Fit In?. Exp. Physiol..

[11] Baker C.J., Min D., Marsh-Wakefield F., Siwan E., Gerofi J., Wang X., Hocking S.L., Colagiuri S., Johnson N.A., Twigg S.M. (2024). Circulating CD31+ Angiogenic T Cells Are Reduced in Prediabetes and Increase with Exercise Training. J. Diabetes Complicat..

[12] Park H.S., Song Y., Lee J.-H., Oh K.-R., Park H., Kang H. (2024). The Role of Exercise in Promoting Lymphangiogenesis and Extracellular Matrix Synthesis in Lymphedema-Induced Tissue Injury. Mol. Biol. Rep..

[13] Verma V.K., Singh V., Singh M.P., Singh S.M. (2009). Effect of Physical Exercise on Tumor Growth Regulating Factors of Tumor Microenvironment: Implications in Exercise-Dependent Tumor Growth Retardation. Immunopharmacol. Immunotoxicol..

[14] Ambrose C. (2015). Muscle Weakness during Aging: A Deficiency State Involving Declining Angiogenesis. Ageing Res. Rev..

[15] Tong X., Chen X., Zhang S., Huang M., Shen X., Xu J., Zou J. (2019). The Effect of Exercise on the Prevention of Osteoporosis and Bone Angiogenesis. Biomed. Res. Int..

[16] Pedrinolla A., Venturelli M., Fonte C., Tamburin S., Di Baldassarre A., Naro F., Varalta V., Giuriato G., Ghinassi B., Muti E. (2020). Exercise Training Improves Vascular Function in Patients with Alzheimer’s Disease. Eur. J. Appl. Physiol..

[17] Vaduganathan M., Mensah G.A., Turco J.V., Fuster V., Roth G.A. (2022). The Global Burden of Cardiovascular Diseases and Risk: A Compass for Future Health. J. Am. Coll. Cardiol..

[18] Van Pelt D.W., Guth L.M., Horowitz J.F. (2017). Aerobic Exercise Elevates Markers of Angiogenesis and Macrophage IL-6 Gene Expression in the Subcutaneous Adipose Tissue of Overweight-to-Obese Adults. J. Appl. Physiol..

[19] Loustau T., Coudiere E., Karkeni E., Landrier J.-F., Jover B., Riva C. (2020). Murine Double Minute-2 Mediates Exercise-Induced Angiogenesis in Adipose Tissue of Diet-Induced Obese Mice. Microvasc. Res..

[20] Disanzo B.L., You T. (2014). Effects of Exercise Training on Indicators of Adipose Tissue Angiogenesis and Hypoxia in Obese Rats. Metabolism.

[21] Olfert I.M., Baum O., Hellsten Y., Egginton S. (2016). Advances and Challenges in Skeletal Muscle Angiogenesis. Am. J. Physiol. Heart Circ. Physiol..

[22] Rosenstein J.M., Krum J.M., Ruhrberg C. (2010). VEGF in the Nervous System. Organogenesis.

[23] Ellison G.M., Waring C.D., Vicinanza C., Torella D. (2012). Physiological Cardiac Remodelling in Response to Endurance Exercise Training: Cellular and Molecular Mechanisms. Heart.

[24] Ramasamy S.K., Kusumbe A.P., Wang L., Adams R.H. (2014). Endothelial Notch Activity Promotes Angiogenesis and Osteogenesis in Bone. Nature.

[25] Min S.Y., Learnard H., Kant S., Gealikman O., Rojas-Rodriguez R., DeSouza T., Desai A., Keaney J.F., Corvera S., Craige S.M. (2019). Exercise Rescues Gene Pathways Involved in Vascular Expansion and Promotes Functional Angiogenesis in Subcutaneous White Adipose Tissue. Int. J. Mol. Sci..

[26] Kuwano M., Fukushi J., Okamoto M., Nishie A., Goto H., Ishibashi T., Ono M. (2001). Angiogenesis Factors. Intern. Med..

[27] Haas T.L., Nwadozi E. (2015). Regulation of Skeletal Muscle Capillary Growth in Exercise and Disease. Appl. Physiol. Nutr. Metab..

[28] Wagenmakers A.J.M., Strauss J.A., Shepherd S.O., Keske M.A., Cocks M. (2016). Increased Muscle Blood Supply and Transendothelial Nutrient and Insulin Transport Induced by Food Intake and Exercise: Effect of Obesity and Ageing. J. Physiol..

[29] Hoier B., Hellsten Y. (2014). Exercise-Induced Capillary Growth in Human Skeletal Muscle and the Dynamics of VEGF. Microcirculation.

[30] Hiscock N., Fischer C.P., Pilegaard H., Pedersen B.K. (2003). Vascular Endothelial Growth Factor mRNA Expression and Arteriovenous Balance in Response to Prolonged, Submaximal Exercise in Humans. Am. J. Physiol. Heart Circ. Physiol..

[31] Nunomiya A., Shin J., Kitajima Y., Dan T., Miyata T., Nagatomi R. (2017). Activation of the Hypoxia-Inducible Factor Pathway Induced by Prolyl Hydroxylase Domain 2 Deficiency Enhances the Effect of Running Training in Mice. Acta Physiol..

[32] Gustafsson T., Knutsson A., Puntschart A., Kaijser L., Nordqvist A.-C.S., Sundberg C.J., Jansson E. (2002). Increased Expression of Vascular Endothelial Growth Factor in Human Skeletal Muscle in Response to Short-Term One-Legged Exercise Training. Pflug. Arch..

[33] Lloyd P.G., Prior B.M., Yang H.T., Terjung R.L. (2003). Angiogenic Growth Factor Expression in Rat Skeletal Muscle in Response to Exercise Training. Am. J. Physiol. Heart Circ. Physiol..

[34] Ruas J.L., White J.P., Rao R.R., Kleiner S., Brannan K.T., Harrison B.C., Greene N.P., Wu J., Estall J.L., Irving B.A. (2012). A PGC-1α Isoform Induced by Resistance Training Regulates Skeletal Muscle Hypertrophy. Cell.

[35] Tamura Y., Kawashima T., Kodama A., Ji R.-C., Itoh Y., Agata N., Kawakami K. (2025). Voluntary Wheel Running Promotes Lymphangiogenesis in Slow-Twitch Muscle in Young Mice. Front. Physiol..

[36] Tomaz da Silva M., Joshi A.S., Koike T.E., Roy A., Mathukumalli K., Sopariwala D.H., Narkar V.A., Kumar A. (2022). Targeted Ablation of Fn14 Receptor Improves Exercise Capacity and Inhibits Neurogenic Muscle Atrophy. FASEB J..

[37] Yao Z., Lafage-Proust M.-H., Plouët J., Bloomfield S., Alexandre C., Vico L. (2004). Increase of Both Angiogenesis and Bone Mass in Response to Exercise Depends on VEGF. J. Bone Miner. Res..

[38] Viboolvorakul S., Niimi H., Wongeak-in N., Eksakulkla S., Patumraj S. (2009). Increased Capillary Vascularity in the Femur of Aged Rats by Exercise Training. Microvasc. Res..

[39] Matsuzaki H., Wohl G.R., Novack D.V., Lynch J.A., Silva M.J. (2007). Damaging Fatigue Loading Stimulates Increases in Periosteal Vascularity at Sites of Bone Formation in the Rat Ulna. Calcif. Tissue Int..

[40] Holstein J.H., Becker S.C., Fiedler M., Scheuer C., Garcia P., Histing T., Klein M., Pohlemann T., Menger M.D. (2011). Exercise Enhances Angiogenesis during Bone Defect Healing in Mice. J. Orthop. Res..

[41] Wang Y., Wan C., Deng L., Liu X., Cao X., Gilbert S.R., Bouxsein M.L., Faugere M.-C., Guldberg R.E., Gerstenfeld L.C. (2007). The Hypoxia-Inducible Factor Alpha Pathway Couples Angiogenesis to Osteogenesis during Skeletal Development. J. Clin. Investig..

[42] Rasmussen P., Brassard P., Adser H., Pedersen M.V., Leick L., Hart E., Secher N.H., Pedersen B.K., Pilegaard H. (2009). Evidence for a Release of Brain-Derived Neurotrophic Factor from the Brain during Exercise. Exp. Physiol..

[43] Fabel K., Fabel K., Tam B., Kaufer D., Baiker A., Simmons N., Kuo C.J., Palmer T.D. (2003). VEGF Is Necessary for Exercise-Induced Adult Hippocampal Neurogenesis. Eur. J. Neurosci..

[44] Stevenson M.E., Miller C.C., Owen H.A., Swain R.A. (2020). Aerobic Exercise Increases Sprouting Angiogenesis in the Male Rat Motor Cortex. Brain Struct. Funct..

[45] Gertz K., Priller J., Kronenberg G., Fink K.B., Winter B., Schröck H., Ji S., Milosevic M., Harms C., Böhm M. (2006). Physical Activity Improves Long-Term Stroke Outcome via Endothelial Nitric Oxide Synthase-Dependent Augmentation of Neovascularization and Cerebral Blood Flow. Circ. Res..

[46] Zhang S., Wu F., Zhan L., Lin W., Liang C., Pang Y., Zhang J., Mu Z. (2022). Exercise Regulates the Lactate Receptor HCAR1 and ERK1/2-PI3K/Akt Pathways to Promote Cerebral Angiogenesis. Iran. J. Public Health.

[47] Fasihiyan M., Nourshahi M., Taheri M., Asadi Y., Pakravan R. (2025). Ischemic Stroke Rehabilitation through Endurance Training of Varying Intensity and Duration in Male Sprague-Dawley Rats. Iran. J. Basic. Med. Sci..

[48] Geng H., Li M., Tang J., Lv Q., Li R., Wang L. (2022). Early Rehabilitation Exercise after Stroke Improves Neurological Recovery through Enhancing Angiogenesis in Patients and Cerebral Ischemia Rat Model. Int. J. Mol. Sci..

[49] Li C., Zhang B., Zhu Y., Li Y., Liu P., Gao B., Tian S., Du L., Bai Y. (2017). Post-Stroke Constraint-Induced Movement Therapy Increases Functional Recovery, Angiogenesis, and Neurogenesis with Enhanced Expression of HIF-1α and VEGF. Curr. Neurovasc. Res..

[50] Li F., Geng X., Huber C., Stone C., Ding Y. (2020). In Search of a Dose: The Functional and Molecular Effects of Exercise on Post-Stroke Rehabilitation in Rats. Front. Cell Neurosci..

[51] Li K., Gao Z.-K., Guo Y.-S., Shen X.-Y., Han Y., Yuan M., Bi X. (2024). Preconditioning Exercise Reduces Brain Damage of Ischemic Stroke in Rats via PI3K-AKT Pathway by Bioinformatic Analysis. Exp. Brain Res..

[52] Tang Y., Zhang Y., Zheng M., Chen J., Chen H., Liu N. (2018). Effects of Treadmill Exercise on Cerebral Angiogenesis and MT1-MMP Expression after Cerebral Ischemia in Rats. Brain Behav..

[53] Yeo H.-S., Lim J.-Y. (2022). Effects of Different Types of Exercise Training on Angiogenic Responses in the Left Ventricular Muscle of Aged Rats. Exp. Gerontol..

[54] Dariushnejad H., Mohammadi M., Ghorbanzadeh V. (2018). Crocin and Voluntary Exercise Promote Heart Angiogenesis through Akt and ERK1/2 Signalling in Type 2 Diabetic Rats. Bratisl. Lek. Listy.

[55] Ghorbanzadeh V., Mohammadi M., Dariushnejad H., Chodari L., Mohaddes G. (2016). Effects of Crocin and Voluntary Exercise, Alone or Combined, on Heart VEGF-A and HOMA-IR of HFD/STZ Induced Type 2 Diabetic Rats. J. Endocrinol. Investig..

[56] Sabzevari Rad R., Shirvani H., Mahmoodzadeh Hosseini H., Shamsoddini A., Samadi M. (2020). Micro RNA-126 Promoting Angiogenesis in Diabetic Heart by VEGF/Spred-1/Raf-1 Pathway: Effects of High-Intensity Interval Training. J. Diabetes Metab. Disord..

[57] Song W., Liang Q., Cai M., Tian Z. (2020). HIF-1α-Induced up-Regulation of microRNA-126 Contributes to the Effectiveness of Exercise Training on Myocardial Angiogenesis in Myocardial Infarction Rats. J. Cell Mol. Med..

[58] Bo W., Ma Y., Feng L., Yu M., Zhang L., Cai M., Song W., Xi Y., Tian Z. (2023). FGF21 Promotes Myocardial Angiogenesis and Mediates the Cardioprotective Effects of Exercise in Myocardial Infarction Mice. J. Appl. Physiol..

[59] Belenguer-Varea A., Avellana-Zaragoza J.A., Inglés M., Cunha-Pérez C., Cuesta-Peredo D., Borrás C., Viña J., Tarazona-Santabalbina F.J. (2023). Effect of Familial Longevity on Frailty and Sarcopenia: A Case-Control Study. Int. J. Environ. Res. Public Health.

[60] Prior S.J., Ryan A.S., Blumenthal J.B., Watson J.M., Katzel L.I., Goldberg A.P. (2016). Sarcopenia Is Associated With Lower Skeletal Muscle Capillarization and Exercise Capacity in Older Adults. J. Gerontol. A Biol. Sci. Med. Sci..

[61] McIntosh M.C., Michel J.M., Godwin J.S., Plotkin D.L., Anglin D.A., Mattingly M.L., Agyin-Birikorang A., Kontos N.J., Baweja H.S., Stock M.S. (2025). Disuse and Subsequent Recovery Resistance Training Affect Skeletal Muscle Angiogenesis-Related Markers Regardless of Prior Resistance Training Experience. J. Appl. Physiol..

[62] Hussain M.A., Al-Omran M., Creager M.A., Anand S.S., Verma S., Bhatt D.L. (2018). Antithrombotic Therapy for Peripheral Artery Disease: Recent Advances. J. Am. Coll. Cardiol..

[63] Lin F., Yao Z., Yu J., Chen X., Chen X., Li Y., Fu J., Cheng Y., Li J., Fang C. (2025). Investigating the Role of Glycolysis in Xuefu Zhuyu Capsule-Promoted Angiogenesis in Endothelial Cells: A Study Based on Network Pharmacology, Molecular Docking, and In Vitro Validation. Pharmaceuticals.

[64] Carmichael E., Reme A.-I.S., Bosco P.J., Ortiz Y.Y., Ramos D.A., Gomez K., Nguyen B.-N., Bornak A., Liu Z.-J., Velazquez O.C. (2025). Biological Regulation of HIF-1α and Its Role in Therapeutic Angiogenesis for Treatment of Ischemic Cardiovascular Disease. Int. J. Mol. Sci..

[65] Shweiki D., Itin A., Soffer D., Keshet E. (1992). Vascular Endothelial Growth Factor Induced by Hypoxia May Mediate Hypoxia-Initiated Angiogenesis. Nature.

[66] Qi C., Song X., Wang H., Yan Y., Liu B. (2022). The Role of Exercise-Induced Myokines in Promoting Angiogenesis. Front. Physiol..

[67] Narkar V.A. (2023). Exercise and Ischemia-Activated Pathways in Limb Muscle Angiogenesis and Vascular Regeneration. Methodist. Debakey Cardiovasc. J..

[68] Sun Z., Yang L., Kiram A., Yang J., Yang Z., Xiao L., Yin Y., Liu J., Mao Y., Zhou D. (2023). FNIP1 Abrogation Promotes Functional Revascularization of Ischemic Skeletal Muscle by Driving Macrophage Recruitment. Nat. Commun..

[69] Zhang S., Guo J., He Y., Su Z., Feng Y., Zhang L., Jun Z., Weng X., Yuan Y. (2025). Roles of lncRNA in the Crosstalk between Osteogenesis and Angiogenesis in the Bone Microenvironment. J. Zhejiang Univ. Sci. B.

[70] Sivan U., De Angelis J., Kusumbe A.P. (2019). Role of Angiocrine Signals in Bone Development, Homeostasis and Disease. Open Biol..

[71] Gómez-Bruton A., Gónzalez-Agüero A., Gómez-Cabello A., Casajús J.A., Vicente-Rodríguez G. (2013). Is Bone Tissue Really Affected by Swimming? A Systematic Review. PLoS ONE.

[72] Zelzer E., McLean W., Ng Y.-S., Fukai N., Reginato A.M., Lovejoy S., D’Amore P.A., Olsen B.R. (2002). Skeletal Defects in VEGF(120/120) Mice Reveal Multiple Roles for VEGF in Skeletogenesis. Development.

[73] Gavin T.P., Drew J.L., Kubik C.J., Pofahl W.E., Hickner R.C. (2007). Acute Resistance Exercise Increases Skeletal Muscle Angiogenic Growth Factor Expression. Acta Physiol..

[74] (2001). NIH Consensus Development Panel on Osteoporosis Prevention, Diagnosis, and Therapy. Osteoporosis Prevention, Diagnosis, and Therapy. JAMA.

[75] Bliuc D., Alarkawi D., Nguyen T.V., Eisman J.A., Center J.R. (2015). Risk of Subsequent Fractures and Mortality in Elderly Women and Men with Fragility Fractures with and without Osteoporotic Bone Density: The Dubbo Osteoporosis Epidemiology Study. J. Bone Miner. Res..

[76] Vogt M.T., Cauley J.A., Kuller L.H., Nevitt M.C. (1997). Bone Mineral Density and Blood Flow to the Lower Extremities: The Study of Osteoporotic Fractures. J. Bone Miner. Res..

[77] You J., Liu M., Li M., Zhai S., Quni S., Zhang L., Liu X., Jia K., Zhang Y., Zhou Y. (2023). The Role of HIF-1α in Bone Regeneration: A New Direction and Challenge in Bone Tissue Engineering. Int. J. Mol. Sci..

[78] Carvalho R.S., Einhorn T.A., Lehmann W., Edgar C., Al-Yamani A., Apazidis A., Pacicca D., Clemens T.L., Gerstenfeld L.C. (2004). The Role of Angiogenesis in a Murine Tibial Model of Distraction Osteogenesis. Bone.

[79] Street J., Bao M., deGuzman L., Bunting S., Peale F.V., Ferrara N., Steinmetz H., Hoeffel J., Cleland J.L., Daugherty A. (2002). Vascular Endothelial Growth Factor Stimulates Bone Repair by Promoting Angiogenesis and Bone Turnover. Proc. Natl. Acad. Sci. USA.

[80] Iadecola C. (2017). The Neurovascular Unit Coming of Age: A Journey through Neurovascular Coupling in Health and Disease. Neuron.

[81] Sweeney M.D., Kisler K., Montagne A., Toga A.W., Zlokovic B.V. (2018). The Role of Brain Vasculature in Neurodegenerative Disorders. Nat. Neurosci..

[82] Swain R.A., Harris A.B., Wiener E.C., Dutka M.V., Morris H.D., Theien B.E., Konda S., Engberg K., Lauterbur P.C., Greenough W.T. (2003). Prolonged Exercise Induces Angiogenesis and Increases Cerebral Blood Volume in Primary Motor Cortex of the Rat. Neuroscience.

[83] Pereira A.C., Huddleston D.E., Brickman A.M., Sosunov A.A., Hen R., McKhann G.M., Sloan R., Gage F.H., Brown T.R., Small S.A. (2007). An in Vivo Correlate of Exercise-Induced Neurogenesis in the Adult Dentate Gyrus. Proc. Natl. Acad. Sci. USA.

[84] Meng X., Wu W., Tang Y., Peng M., Yang J., Yuan S., Hu Z., Liu W. (2024). Lactate/Hydroxycarboxylic Acid Receptor 1 in Alzheimer’s Disease: Mechanisms and Therapeutic Implications-Exercise Perspective. Mol. Neurobiol..

[85] Pahlavani H.A. (2023). Exercise Therapy to Prevent and Treat Alzheimer’s Disease. Front. Aging Neurosci..

[86] Ding Y.-H., Li J., Zhou Y., Rafols J.A., Clark J.C., Ding Y. (2006). Cerebral Angiogenesis and Expression of Angiogenic Factors in Aging Rats after Exercise. Curr. Neurovasc. Res..

[87] Grinberg L.T., Heinsen H. (2010). Toward a Pathological Definition of Vascular Dementia. J. Neurol. Sci..

[88] Bonthius D.J., Karacay B., Dai D., Pantazis N.J. (2003). FGF-2, NGF and IGF-1, but Not BDNF, Utilize a Nitric Oxide Pathway to Signal Neurotrophic and Neuroprotective Effects against Alcohol Toxicity in Cerebellar Granule Cell Cultures. Brain Res. Dev. Brain Res..

[89] Zang Q., Wang S., Qi Y., Zhang L., Huang C., Xiu Y., Zhou C., Luo Y., Jia G., Li S. (2023). Running Exercise Improves Spatial Learning and Memory Ability and Enhances Angiogenesis in the Cerebral Cortex via Endogenous Nitric Oxide. Behav. Brain Res..

[90] Maida C.D., Norrito R.L., Daidone M., Tuttolomondo A., Pinto A. (2020). Neuroinflammatory Mechanisms in Ischemic Stroke: Focus on Cardioembolic Stroke, Background, and Therapeutic Approaches. Int. J. Mol. Sci..

[91] Fuertes-Kenneally L., Baladzhaeva S., Manresa-Rocamora A., Sempere-Ruiz N., Sanz-Rocher A., Blasco-Peris C., Sarabia J.M. (2026). Effect of Exercise Modality and Intensity on Endothelial Function in Patients with Cardiovascular Disease: A Systematic Review and Network Meta-Analysis. Eur. J. Prev. Cardiol..

[92] Seyydi S.M., Tofighi A., Rahmati M., Tolouei Azar J. (2022). Exercise and Urtica Dioica Extract Ameliorate Mitochondrial Function and the Expression of Cardiac Muscle Nuclear Respiratory Factor 2 and Peroxisome Proliferator-Activated Receptor Gamma Coactivator 1-Alpha in STZ-Induced Diabetic Rats. Gene.

[93] Andersen M.J., Olson T.P., Melenovsky V., Kane G.C., Borlaug B.A. (2015). Differential Hemodynamic Effects of Exercise and Volume Expansion in People with and without Heart Failure. Circ. Heart Fail..

[94] Anversa P., Levicky V., Beghi C., McDonald S.L., Kikkawa Y. (1983). Morphometry of Exercise-Induced Right Ventricular Hypertrophy in the Rat. Circ. Res..

[95] Anversa P., Capasso J.M. (1991). Loss of Intermediate-Sized Coronary Arteries and Capillary Proliferation after Left Ventricular Failure in Rats. Am. J. Physiol..

[96] Liu X., Oliver G. (2023). The Lymphatic Vasculature in Cardiac Development and Ischemic Heart Disease. Circ. Res..

[97] Olamazadeh M.H., Esfarjani F., Marandi S.M., Zamani S., Rarani F.Z., Sharifi M. (2025). The Heart Tissue Molecular Response to Resistance Training in Comparison to Irisin Injection: A Focus on VEGF Gene/Protein Expression and Correlations with Serum Irisin Levels. Int. J. Prev. Med..

[98] Ma Z., Ding M., Hu Y., Mou C., Xun W., Cen Y., Sun J., Bi R., Qui Y., Huo K. (2025). TESC Activates the C-SRC/IGF1Rβ/PI3K/AKT/VEGFA Signaling Pathway to Promote Myocardial Angiogenesis in Cardiac Hypertrophy Induced by an 8-Week Swimming Exercise Program. Gene.

[99] Sylviana N., Goenawan H., Susanti Y., Lesmana R., Megantara I., Setiawan S. (2022). Effect of Different Intensities Aerobic Exercise to Cardiac Angiogenesis Regulation on Wistar Rats. Pol. J. Vet. Sci..

[100] Hinkel R., Howe A., Renner S., Ng J., Lee S., Klett K., Kaczmarek V., Moretti A., Laugwitz K.-L., Skroblin P. (2017). Diabetes Mellitus-Induced Microvascular Destabilization in the Myocardium. J. Am. Coll. Cardiol..

[101] Erekat N.S., Al-Jarrah M.D., Al Khatib A.J. (2014). Treadmill Exercise Training Improves Vascular Endothelial Growth Factor Expression in the Cardiac Muscle of Type I Diabetic Rats. Cardiol. Res..

[102] Chodari L., Mohammadi M., Ghorbanzadeh V., Dariushnejad H., Mohaddes G. (2016). Testosterone and Voluntary Exercise Promote Angiogenesis in Hearts of Rats with Diabetes by Enhancing Expression of VEGF-A and SDF-1a. Can. J. Diabetes.

[103] Connelly K.A., Zhang Y., Advani A., Advani S.L., Thai K., Yuen D.A., Gilbert R.E. (2013). DPP-4 Inhibition Attenuates Cardiac Dysfunction and Adverse Remodeling Following Myocardial Infarction in Rats with Experimental Diabetes. Cardiovasc. Ther..

[104] Yazdani F., Shahidi F., Karimi P. (2020). The Effect of 8 Weeks of High-Intensity Interval Training and Moderate-Intensity Continuous Training on Cardiac Angiogenesis Factor in Diabetic Male Rats. J. Physiol. Biochem..

[105] Fish J.E., Santoro M.M., Morton S.U., Yu S., Yeh R.-F., Wythe J.D., Ivey K.N., Bruneau B.G., Stainier D.Y.R., Srivastava D. (2008). miR-126 Regulates Angiogenic Signaling and Vascular Integrity. Dev. Cell.

[106] Sarlak Z., Eidi A., Ghorbanzadeh V., Moghaddasi M., Mortazavi P. (2023). miR-34a/SIRT1/HIF-1α Axis Is Involved in Cardiac Angiogenesis of Type 2 Diabetic Rats: The Protective Effect of Sodium Butyrate Combined with Treadmill Exercise. Biofactors.

[107] Chodari L., Dariushnejad H., Ghorbanzadeh V. (2019). Voluntary Wheel Running and Testosterone Replacement Increases Heart Angiogenesis through miR-132 in Castrated Diabetic Rats. Physiol. Int..

[108] Boateng S., Sanborn T. (2013). Acute Myocardial Infarction. Dis. Mon..

[109] Wang B., Zhou R., Wang Y., Liu X., Shou X., Yang Y., Yang C., Tong Q., Mao G., Wu Q. (2020). Effect of High-Intensity Interval Training on Cardiac Structure and Function in Rats with Acute Myocardial Infarct. Biomed. Pharmacother..

[110] Wu G., Rana J.S., Wykrzykowska J., Du Z., Ke Q., Kang P., Li J., Laham R.J. (2009). Exercise-Induced Expression of VEGF and Salvation of Myocardium in the Early Stage of Myocardial Infarction. Am. J. Physiol. Heart Circ. Physiol..

[111] Huang H., Huang G., Li R., Wei L., Yuan Z., Huang W. (2025). Exercise Training After Myocardial Infarction Enhances Endothelial Progenitor Cells Function via NRG-1 Signaling. Cardiovasc. Toxicol..

[112] Xi Y., Hao M., Liang Q., Li Y., Gong D.-W., Tian Z. (2021). Dynamic Resistance Exercise Increases Skeletal Muscle-Derived FSTL1 Inducing Cardiac Angiogenesis via DIP2A-Smad2/3 in Rats Following Myocardial Infarction. J. Sport. Health Sci..

[113] Marques R.F., de Andrade M.S., Ferreira A.C., Dias C.J.M., de Jesus Silva Soares Junior N., Filho C.A.A.D., Ribeiro R.M. (2025). The Role of Physical Exercise in Modulating microRNAs Expression in Acute Myocardial Infarction: A Review. Mol. Cell Biochem..

[114] Saati-Zarei A., Damirchi A., Tousi S.M.T.R., Babaei P. (2023). Myocardial Angiogenesis Induced by Concurrent Vitamin D Supplementation and Aerobic-Resistance Training Is Mediated by Inhibiting miRNA-15a, and miRNA-146a and Upregulating VEGF/PI3K/eNOS Signaling Pathway. Pflug. Arch..

[115] Chen C.M. (2008). Overview of Obesity in Mainland China. Obes. Rev..

[116] Von Bank H., Kirsh C., Simcox J. (2021). Aging Adipose: Depot Location Dictates Age-Associated Expansion and Dysfunction. Ageing Res. Rev..

[117] May F.J., Baer L.A., Lehnig A.C., So K., Chen E.Y., Gao F., Narain N.R., Gushchina L., Rose A., Doseff A.I. (2017). Lipidomic Adaptations in White and Brown Adipose Tissue in Response to Exercise Demonstrate Molecular Species-Specific Remodeling. Cell Rep..

[118] Lehnig A.C., Dewal R.S., Baer L.A., Kitching K.M., Munoz V.R., Arts P.J., Sindeldecker D.A., May F.J., Lauritzen H.P.M.M., Goodyear L.J. (2019). Exercise Training Induces Depot-Specific Adaptations to White and Brown Adipose Tissue. iScience.

[119] Sepa-Kishi D.M., Ceddia R.B. (2016). Exercise-Mediated Effects on White and Brown Adipose Tissue Plasticity and Metabolism. Exerc. Sport. Sci. Rev..

[120] Arderiu G., Lambert C., Ballesta C., Moscatiello F., Vilahur G., Badimon L. (2020). Cardiovascular Risk Factors and Differential Transcriptomic Profile of the Subcutaneous and Visceral Adipose Tissue and Their Resident Stem Cells. Cells.

[121] Xue Y., Petrovic N., Cao R., Larsson O., Lim S., Chen S., Feldmann H.M., Liang Z., Zhu Z., Nedergaard J. (2009). Hypoxia-Independent Angiogenesis in Adipose Tissues during Cold Acclimation. Cell Metab..

[122] Corvera S., Solivan-Rivera J., Yang Loureiro Z. (2022). Angiogenesis in Adipose Tissue and Obesity. Angiogenesis.

[123] Divoux A., Clément K. (2011). Architecture and the Extracellular Matrix: The Still Unappreciated Components of the Adipose Tissue. Obes. Rev..

[124] Ioannidou A., Fisher R.M., Hagberg C.E. (2022). The Multifaceted Roles of the Adipose Tissue Vasculature. Obes. Rev..

[125] Fitzgibbons T.P., Kogan S., Tran K.-V. (2025). Vascular-Adipose Crosstalk: Angiogenesis and Adipose Tissue Remodeling. Circ. Res..

[126] Wang R., Sun Q., Wu X., Zhang Y., Xing X., Lin K., Feng Y., Wang M., Wang Y., Wang R. (2022). Hypoxia as a Double-Edged Sword to Combat Obesity and Comorbidities. Cells.

[127] Kolahdouzi S., Talebi-Garakani E., Hamidian G., Safarzade A. (2019). Exercise Training Prevents High-Fat Diet-Induced Adipose Tissue Remodeling by Promoting Capillary Density and Macrophage Polarization. Life Sci..

[128] Armulik A., Genové G., Betsholtz C. (2011). Pericytes: Developmental, Physiological, and Pathological Perspectives, Problems, and Promises. Dev. Cell.

[129] AlQabandi Y., Nandula S.A., Boddepalli C.S., Gutlapalli S.D., Lavu V.K., Abdelwahab Mohamed Abdelwahab R., Huang R., Potla S., Bhalla S., Hamid P. (2022). Physical Activity Status and Diabetic Retinopathy: A Review. Cureus.

